# Incidentally Discovered Extranodal Marginal Zone B-Cell Lymphoma of Mucosa-Associated Lymphoid Tissue in the Colon

**DOI:** 10.1155/2017/1505706

**Published:** 2017-12-04

**Authors:** Raja Chandra Chakinala, Khwaja F. Haq, Jonathan E. Barsa, Shantanu Solanki, Lavneet Chawla, Muhammad Ali Khan, Taliya Farooq, Beth Schorr-Lesnick

**Affiliations:** ^1^New York Medical College, Westchester Medical Center, New York, NY, USA; ^2^Division of Gastroenterology and Hepatobiliary Diseases, New York Medical College, Westchester Medical Center, New York, NY, USA; ^3^Division of Gastroenterology, University of Tennessee Health Science Center, Memphis, TN, USA; ^4^Department of Pathology, New York Medical College, Westchester Medical Center, New York, NY, USA

## Abstract

We present a case of colonic mucosa-associated lymphoid tissue (MALT) lymphoma in a 62-year-old woman diagnosed after a positive test for fecal occult blood.

## 1. Introduction

MALT lymphoma, also referred to as extranodal marginal zone B-cell lymphoma, is a low-grade malignant non-Hodgkin lymphoma that develops in mucosa-associated lymphoid tissue (MALT) [1]. Although a relatively rare disease, MALT lymphoma accounts for approximately 7% to 8% of all non-Hodgkin lymphomas (NHLs) [1] and is one of the two most common types of gastrointestinal NHLs, with diffuse large B-cell (DLBC) NHL being the other one [2, 3]. The gastrointestinal (GI) tract is the most common site for extranodal lymphomas, with stomach being the most commonly affected GI tract site. However, MALT lymphomas can arise at any extranodal site in various epithelial tissues, such as the salivary gland, lung, liver, thyroid gland, breast, and dura mater [1, 2, 4, 5].

Colonic MALT lymphoma, unlike gastric MALT lymphoma, is very rare (2.5% versus 60–75%) [3, 6]. The presentation of colonic MALT lymphoma is diverse, ranging from an incidental finding on colonoscopy to massive GI bleeding [2, 6–9]. We present such a case occurring in a 62-year-old woman diagnosed after a positive test for fecal occult blood.

## 2. Case Presentation

A 62-year-old female with a history of recurrent urinary tract infections, nephrolithiasis, Bell's palsy, and vulvar intraepithelial neoplasia grade 2 was found to have a decrease in hematocrit during a routine follow-up visit at our clinic. Iron studies were suggestive of iron deficiency anemia, and further workup showed a positive fecal occult blood test. She reported no complaints of abdominal pain, hematemesis, melena, hematochezia, or weight loss. The patient denied change in appetite or bowel habits. Physical examination revealed no palpable lymph nodes, no petechiae, no hepatomegaly, and no external hemorrhoids. She had no signs of autoimmune disease.

**Figure 1:**
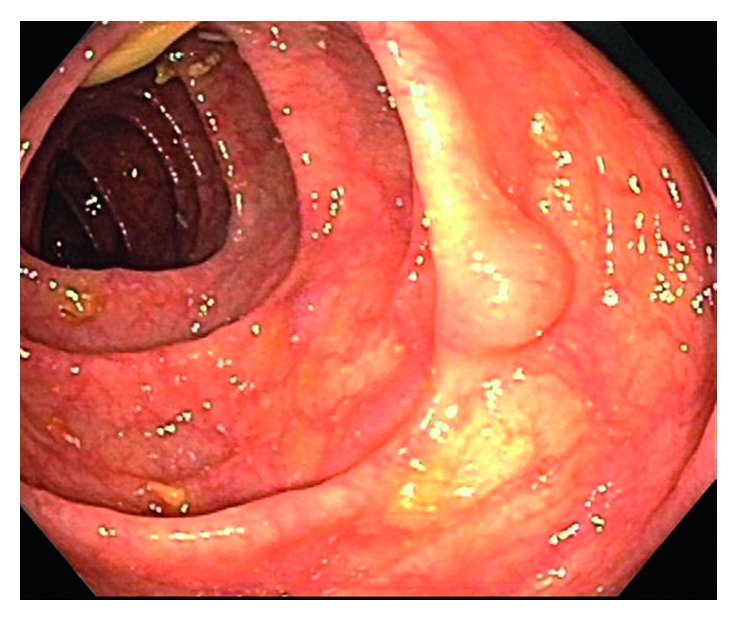
Colonoscopy shows a 10 mm sessile polyp with smooth edges (arrow) in the transverse colon.

**Figure 2:**
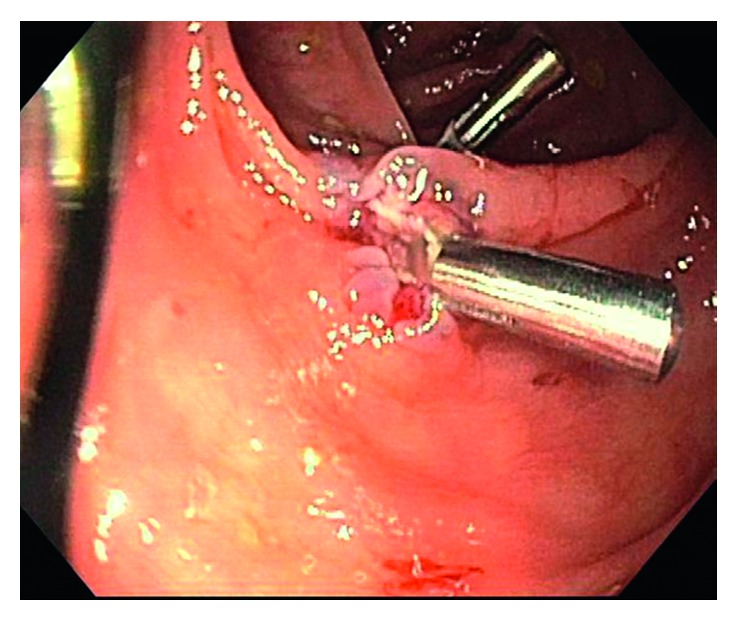
The polyp was removed using snare cautery, and two clips were placed at the polypectomy site to help prevent bleeding.

**Figure 3:**
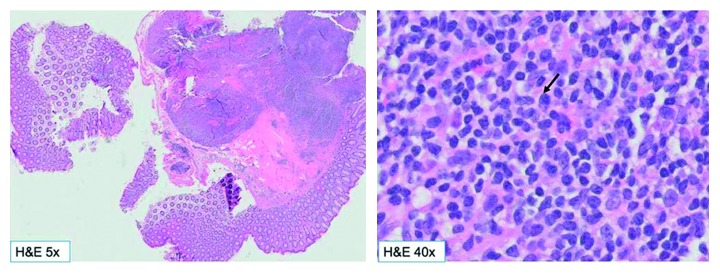
The H&E sections show proliferation of atypical lymphoid cells involving the colonic mucosa and submucosa, forming multifocal large lymphoid aggregates. H&E section on 40x shows lymphoid cells that are small in size and have scant to moderate cytoplasm. Admixed with them are scattered plasma cells (arrow).

Colonoscopy was significant for sigmoid diverticulosis and a smooth, sessile 10 mm polyp in the transverse colon, which was removed with hot snare (Figures 1 and 2). Pathological examination of the polyp showed colonic mucosa with atypical lymphoid aggregates, suspicious for low-grade lymphoproliferative disorder (Figure 3). Immunohistochemical staining of the atypical lymphocytes was positive for CD20, bcl2, and CD43 but negative for CD3, CD5, CD10, cyclinD1, and CD21. Molecular studies were consistent with MALT lymphoma. Upper endoscopy revealed diffuse chronic active gastritis, positive for *Helicobacter pylori* (*H. pylori*) by immunohistochemical staining, but was negative for dysplasia, metaplasia, or malignancy. The patient was treated for *H. pylori* gastritis with triple therapy (Biaxin, amoxicillin, and omeprazole). She underwent further workup to rule out any distant disease with computerized tomography of chest, abdomen, and pelvis that showed no evidence of lymphoma, soft tissue mass, or adenopathy within the chest, abdomen, or pelvis. Bone marrow biopsy of her right iliac crest showed normocellular marrow negative for lymphoma.

She had ongoing follow-up at hematology and GI clinics. Repeat colonoscopy showed a tubular adenoma in the ascending colon and a polypoid fragment of colonic mucosa with benign lymphoid aggregate in the rectum, but no tumor recurrence.

## 3. Discussion

MALT lymphoma, a term first coined by Issacson and Wright in 1983, is a low-grade B-cell lymphoma that occurs in a variety of extranodal organs [10]. It is now classified as extranodal B-cell lymphoma of MALT type. These are mainly seen in adults. A slight female predominance has been reported [11, 12]. The most common site for MALT lymphoma is the stomach; lymphomas in colon and rectum are rare (<10%) [13]. Most colonic MALT lymphomas have been described as a single polypoid lesion or as a submucosal tumor, but multiple polypoid lesions or slight mucosal thickening have also been reported [6, 10]. Our patient had a biopsy positive for marginal zone B-cell lymphoma in the transverse colon, which presented as a single, smooth, sessile 10 mm polyp. Patients can present with symptoms of abdominal pain, weight loss, and/or decreased appetite. Alternatively, they may be diagnosed after they are incidentally found to have a mass at the site of involvement during upper endoscopy or cross-sectional imaging [5, 14]. Our patient presented with iron deficiency anemia and positive stool for occult blood which is not explained by the lesion described. Further workup for these indications is ongoing with CT enterography planned. There is no standardized treatment for management of colonic MALT lymphoma [10]. Successful treatment of colonic MALT lymphoma by eradication of *H. pylori* has been reported, even in cases when *H. pylori* testing was negative [15], and the reason for regression of the lesion was speculated to be elimination of pathogenic bacteria other than *H. pylori* by antibiotics. Upper endoscopy performed in our patient revealed chronic active gastritis, and it was positive for *H. pylori* by immunohistochemical staining. The patient was treated for *H. pylori* gastritis with triple therapy. Limited stage nongastric MALT lymphomas can be treated with locoregional radiation therapy [16, 17]. Although radiotherapy can be effective in providing local disease control even for some patients with disseminated disease [18], there is no clear consensus as to whether radiation is more or less effective than systemic therapy in MALT lymphomas at different locations. Hence, the decision of radiation versus chemotherapy can be made based on individual patient's profile and the center's experience [19]. In advanced stage disease when systemic treatment is needed, chemotherapy and/or immunotherapy with anti-CD20 monoclonal antibodies can be considered [19, 20]. Surgical resection is recommended when a colorectal MALT lymphoma does not respond to eradication therapy or chemotherapy, provided it is localized without dissemination [21]. The overall prognosis of nongastric MALT is poor with five-year and 10-year overall survival rates of approximately 50 and 20 percent, respectively, with disseminated lymphoma as the usual cause of death [22, 23]. Given the poor prognosis, a periodic clinical monitoring of these patients is recommended. Our patient has an ongoing follow-up at both gastroenterology and hematology clinics with a plan for surveillance colonoscopy every 6 months.
